# Stanniocalcin-2 significantly promotes colorectal cancer progression by regulating cancer cell proliferation and invasion

**DOI:** 10.7150/jca.101892

**Published:** 2025-06-12

**Authors:** Fang Li, Zihao Liu, Kaibin Huang, Shunkai Ding, Chang Zhu, Yuxiang Fu, Xiao Sun, Shipai Zhang, Rui Zhang, Zhipeng Jiang, Keli Zhong, Qijun Zheng

**Affiliations:** 1 Department of Gastrointestinal Surgery, Shenzhen People's Hospital (The Second Clinical Medical College, Jinan University, The First Affiliated Hospital, Southern University of Science and Technology), Shenzhen, Guangdong, China, 518020.; 2 Department of Cardiovascular Surgery, Shenzhen People's Hospital (The Second Clinical Medical College, Jinan University; The First Affiliated Hospital, Southern University of Science and Technology), Shenzhen, Guangdong, China.; 3 Department of Breast Surgery, The Third Affiliated Hospital of Kunming Medical University, Yunnan Cancer Hospital, Peking University Cancer Hospital Yunnan Hospital, Kunming, Yunnan, China.

**Keywords:** STC2, colorectal cancer, pan-cancer, tumorigenesis, oncogenesis

## Abstract

Colorectal cancer (CRC) remains a leading cause of cancer-related mortality worldwide. Our study delves into the molecular intricacies of CRC by analyzing gene expression profiles across multiple datasets, revealing significant gene alterations that distinguish CRC from normal tissues. We identified Stanniocalcin-2 (STC2) as a key regulator in CRC, associated with poor prognosis, survival outcomes and cancer cell proliferation or invasion. Through comprehensive data mining of the Gene Expression Omnibus (GEO), the European Bioinformatics Institute (EMBL-EBI), and The Cancer Genome Atlas (TCGA), we emphasized the role of STC2 in tumorigenesis. Our pan-cancer analysis established STC2's involvement in various cancer types, underscoring its potential as a universal biomarker. Additionally, we performed experimental research and found STC2 is significantly upregulated in CRC tissue and can promote CRC progression by regulating cancer cell invasion and proliferation. This study provides valuable insights into the oncogenic role of STC2, proposing it as a promising target for therapeutic intervention and a marker for aggressive cancer phenotypes.

## Introduction

Colorectal cancer (CRC) represents a significant challenge to public health globally, ranking as the third most common cancer and the second leading cause of cancer-related deaths worldwide^1^, ^2^. Therefore, there is the urgent need for enhanced diagnostic and therapeutic strategies to improve patient outcomes of CRC patients. The intricate associations between genetic, environmental, and lifestyle factors contribute to the pathogenesis of CRC, yet the molecular mechanisms underlying its tumorigenesis and progression remain incompletely understood^3^, ^4^. Overall, new biomarkers that can accurately predict survival and therapeutic targets that can improve prognosis in CRC are needed.

Stanniocalcin-2 (STC2), a secreted glycoprotein implicated in various physiological processes, including calcium and phosphate homeostasis, has emerged as a molecule of interest in cancer research^5^, ^6^. Accumulating evidence suggests that STC2 plays a functional role in cancer development and progression, acting through mechanisms that influence cell proliferation, migration, invasion, and the tumor microenvironment (TME)^7^. This has been demonstrated across several cancers, including head and neck squamous cell carcinoma^8^, ^9^, gastric cancer^10^, and hepatocellular carcinoma^11^, ^12^. Recent studies have highlighted the prognostic value of STC2, associating its overexpression with poor survival outcomes in several cancer types. Moreover, STC2's involvement in modulating the immune landscape within the TME suggests its potential as a target for immunotherapy, offering new avenues for cancer treatment.

The potential involvement of STC2 in colorectal cancer has begun to draw attention. Only few studies reported the role of STC2 in colorectal cancer. A single center study reveals that the high expression of STC2 is associated with poor survival of CRC patient^13^. Another study indicates that STC2 associates with epithelium-mescenchymal transition of CRC cells^14^. However, the exact role of STC2 in CRC remains to be fully elucidated. Given the pressing need for better diagnostic markers and therapeutic targets in CRC, this study aims to explore the relationship between STC2 expression and colorectal cancer comprehensively. We seek to assess its potential as a prognostic marker and therapeutic target.

In this study, we comprehensively integrate samples from The Cancer Genome Atlas (TCGA) database and the Gene Expression Omnibus (GEO) datasets to perform large scale data mining. Through differentially expressed genes and prognosis-related genes analysis, we further screened out a key gene, STC2, with the highest risk for validation. We investigate the relationship between STC2, patient prognosis, immune infiltration status and tumor mutation burden. The expression of STC2 was confirmed by immunohistochemistry and the functional roles of STC2 were detected in CRC cells.

## Materials and Methods

### Databases and samples

The RNA sequencing data, somatic mutation, copy number variation (CNV), and clinical data for CRC were retrieved from TCGA database (https://portal.gdc.cancer.gov/). All CRC samples from TCGA were enrolled including 480 colorectal cancer tissue samples and 41 adjacent normal colorectal tissue samples. For validation, six independent cohorts were obtained from the GEO datasets (https://www.ncbi.nlm.nih.gov/geo/), GSE74602, GSE89393, GSE100243, GSE166427, GSE143939 and GSE144259. Additionally, RNA sequencing data of 33 different types of tumor and matched adjacent normal tissues from TCGA were downloaded from the TCGA data portal (https://portal.gdc.cancer.gov/). The relative expression data of STC2, along with its clinical associations, were analyzed across various cancer types. Basically, the FPKM of mRNA expression data was obtained from TCGA data portal (https://portal.gdc.cancer.gov/) and the FPKM data was transformed into TPM data. The immune infiltration score of different types of cells was downloaded from The Immune Landscape of Cancer^15^, The immune scores, stromal scores and ESTIMATE scores were calculated using the ESTIMATE R package (v1.0.13) based on the gene relative expression of TPM matrix^16^. A total of 39 colorectal cancer tissue samples and 10 adjacent normal colorectal tissue samples were enrolled between 1 June 2019 and 31 October 2022 in Shenzhen People's hospital. Prior to tissue collection, patients were well informed and the hospital's ethics committee had approval this study. Their paraffin-embedded tissue samples were collected for immunohistochemistry. Patients who received chemotherapy or radiotherapy prior to surgery were excluded.

### Cell culture and siRNA transfection

Human colorectal cancer cell lines DLD1, HCT116 and HIEC6 were purchased from ATCC. The three cell lines were certificated by short tandem repeat (STR) and cultured less than 6 months. DLD1 and HCT116 were grown in Dulbecco's modified Eagle medium which contains 10% fetal bovine serum. HIEC6 were grown in Roswell Park Memorial Institute 1640 medium which contains 10% fetal bovine serum. Colorectal cancer cells (10^5^ per well) were seeded in a 6-well plate. Cells were cultured and transfected from 30-50% confluence. Two siRNAs targeting different sites of STC2 (siRNA-1, sense: 5'-GUGGAGAUGAUCCAUUUCA-3', anti-sense: 5'-UGAAAUGGAUCAUCUCCAC-3'; siRNA-2, sense: 5'-GUGGAGAUGAUCCAUUUCA-3', anti-sense: 5'-UGAAAUGGAUCAUCUCCAC-3', Genepharma, Shanghai, China) were, respectively, suspended in Opti-MEM and mixed with lipo3000. The mixture was added to plates and cultured for 48 h. After transfection, cells were harvested for further experiments.

### Differentially expressed gene analysis

For microarray data, differentially expressed genes (DEGs) were identified using the "Limma" package in R software. For RNA sequencing data, DEGs were analyzed using “DESeq2” package in R software. The criteria for significantly upregulated genes were set as *P* value < 0.05 and the log_2_(fold change) > 1.5 and significantly downregulated genes were set as *P* value < 0.05 and the log_2_(fold change) < -1.5 Volcano plots and heatmaps were visualized through "ggplot2" package in R software.

### Gene Set Enrichment Analysis (GSEA)

Kyoto Encyclopedia of Genes and Genomes (KEGG) pathway analysis was performed. The differentially expressed genes were compared between tumor samples and adjacent normal tissue samples. Differentially expressed genes were set as *P* value < 0.05 and the absolute fold change > 2. DEGs were used as input for KEGG analysis through “GSEABase”, “fgsea” and “clusterProfiler” packages in R software. The results of KEGG were visualized using the "enrichplot" in R.

### Genetic alteration analysis

To analyze tumor mutational burden (TMB) in colorectal cancer, “TCGAbiolinks” was used to download the single-nucleotide variation (SNV) and insertion/indel data including missense, frameshift, non-sense, non-stop and splice site from TCGA. TMB was defined as the number of somatic mutations in per megabase base of genome region. For microsatellite instability (MSI) analysis, the MSI status was extracted from TCGA data portal (https://portal.gdc.cancer.gov/). The TCGA consortium's evaluation of MSI employed a combination of four mononucleotide including BAT25, BAT26, BAT40 and TGFBRII and three dinucleotide repeats including D2S123, D5S346 and D17S250. The mutation status, amplification status and deep deletion status were identified by cBioPortal (https://www.cbioportal.org/) across all types of cancers.

### RNA isolation and quantitative real-time PCR

Total RNA was extracted using TRIzol reagent (Life Technologies, USA) according to the manufacturer's protocols. Complementary DNA was synthesized using the RT reagent kit (Takara, Tokyo, Japan) and the qPCR was performed using SYBR green reagent (Vazyme, China). The relative fold-change of gene expression was normalized to ACTB and was calculated by the 2-ΔΔCt method. Primers and probes are listed as follow: ACTB F: 5'-TCATGAAGTGTGACGTGGACATC-3' , R: 5'-CAGGAGGAGCAATGATCTTGATCT-3'; STC2, F: 5'-ATGCTACCTCAAGCACGACC-3'; R: 5'-TCTGCTCACACTGAACCTGC-3'.

### Transwell assay

Migration and invasion ability of colorectal cancer cells were examined using Boyden chambers. For invasion assay, the chambers of the 8 μm inserts were coated with Matrigel. Colorectal cancer cells (10^5^ cells/well) were suspended with serum-free culture medium and plated in the upper chambers of the inserts (uncoated inserts for migration assay and coated inserts for invasion assay). After cultured for 48 hours at 5% CO_2_ 37° C, cells which did not migrate or invade were removed. The inserts then were fixed with 4% formaldehyde, stained with crystal violet and counted cells per field under microscope.

### Colony formation assay

After transfection, a total of 2000 cells were seeded in six-well plates and were incubated at 5% CO_2_ 37° C for 2 weeks. The colonies were fixed with 4% formaldehyde, stained with crystal violet and counted. Independent experiments were repeated for three times.

### CCK8 assay

CCK8 assay (Dojindo Laboratories, Japan) was used to assess cell proliferation state. Cells (3 × 10^3^ per well) were seeded into 96-well plates and were incubated at 5% CO_2_ 37° C. For each assay, 10% of CCK8 reagent was added in each well and incubated at 5% CO_2_, 37° C. Absorbance at 450nm was measured.

### Immunohistochemistry

Immunohistochemistry (IHC) was performed according to following procedures. Formalin-fixed samples were deparaffinized with 100% xylene and rehydrated with different graded ethanol. After being treated with 3% H_2_O_2_, the specimen was treated with sodium citrate to retrieve antigen. After being incubated with 10% serum, the slides were treated with anti-STC2 antibodies (1:100 dilutions, Abcam, Cat log#ab261915) overnight and isotype-matched IgG was used as a negative control. The slides were washed with PBS and incubated with HRP-linked secondary antibodies. Then standard HRP detection procedure was used to detect the slides. Cells with cytoplasmic staining were regarded as positive cells. The percentage of STC2 positive cells in the whole fields of view was calculated to quantify the expression level of STC2 in each slide.

### Survival analysis

Disease-specific survival (DSS), disease-free interval (DFI), and progression-free interval (PFI), were obtained from the TCGA consortium's Clinical Data Resource from publication^17^. Survival rates and curves were determined by the Kaplan-Meier method, and the comparison of survival differences was evaluated by the log-rank test.

### Statistical analysis

The paired and unpaired Student's *t* test were employed for comparing two groups with normally distributed variables, and Mann-Whitney U test was employed for comparing two groups with non-normally distributed variables respectively. For multi-group comparisons, ANOVA and Kruskal-Wallis tests served as parametric and non-parametric options. Kaplan-Meier method was used to compare the survival differences between two groups by the log-rank test. Univariate and multivariate COX regression analysis was used for analyzing hazard ration of STC2 and survival data. Graphpad Prism version (GraphPad, San Diego, USA) and SPSS version 20.0 (Chicago, IL, USA) were used for statistical analysis. *P* values < 0.05 were considered statistically significant. All statistical tests were two-tail.

Supplementary data is available upon reasonable request.

## Results

### Pan-cancer analysis indicated STC2 involved in multiple cancer prognosis

**Figure 1:**
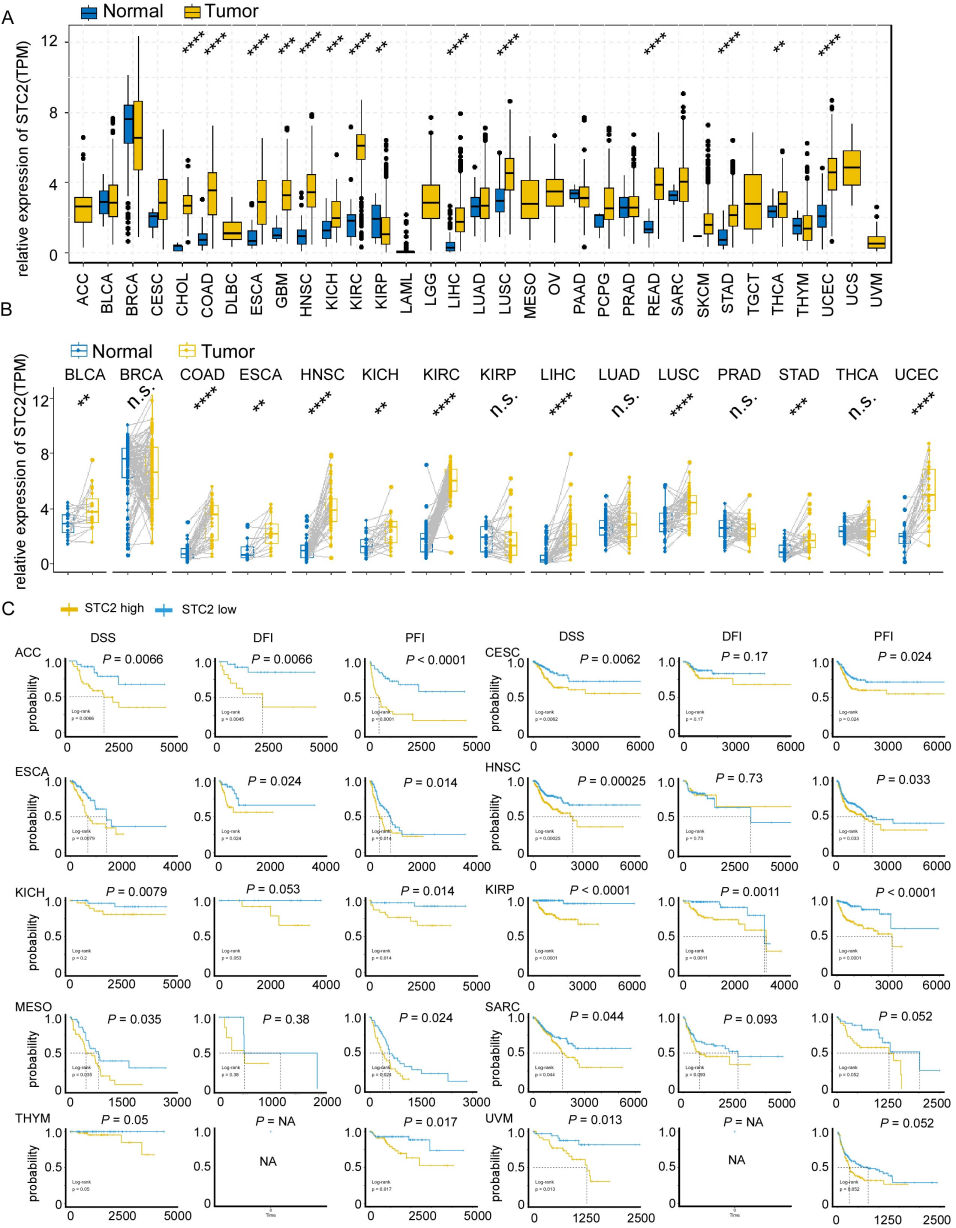
Expression and prognostic significance of STC2 across various cancer types. A. Box plots display the relative expression of STC2 in different cancer types and adjacent normal tissues. B. Box plots display the relative expression of STC2 in cancer tissue and paired adjacent normal tissues. C. Kaplan-Meier survival curves present disease-specific survival (DSS), disease-free interval (DFI), and progression-free interval (PFI), for different types of cancer. (yellow curve for high STC2 and blue curve for low STC2). Abbreviations: adrenocortical cancer (ACC), bladder cancer (BLCA), breast cancer (BRCA), cervical cancer (CESC), cholangiocarcinoma (CHOL), colon cancer (COAD), large B-cell lymphoma (DLBC), esophageal cancer (ESCA), glioblastoma (GBM), head and neck cancer (HNSC), kidney chromophobe cancer (KICH), kidney clear cell carcinoma (KIRC), kidneynpapillary cell carcinoma (KIRP), acute myeloid leukemia (LAML), lower grade glioma (LGG), liver cancer (LIHC), lung adenocarcinoma (LUAD), lung squamous cell carcinoma (LUSC), mesothelioma (MESO), ovarian cancer (OV), pancreatic cancer (PAAD), pheochromocytoma and paraganglioma (PCPG), prostate cancer (PRAD), rectal cancer (READ), sarcoma (SARC), melanoma (SKCM), stomach cancer (STAD), testicular cancer (TGCT), thyroid cancer (THCA), thymoma (THYM), endometrioid cancer (UCEC), uterine carcinosarcoma (UCS), ocular melanomas (UVM). ****, *P* < 0.0001; ***, *P* < 0.001; **, *P* < 0.01; *, *P* < 0.05; ns: not significant. 'NA' indicates that data for a particular metric was not available for that cancer type.

Since previous study revealed STC2 may function in neck squamous cell carcinoma, gastric cancer, and hepatocellular carcinoma, we tend to perform pan-cancer analysis to uncover the oncogenic roles of STC2. Box plots of normal and tumor tissues across 20 different cancer types were shown in Figure 1A. It is found that elevated expression of STC2 in tumor tissues is significant in the majority of cancer types, particularly in colon adenocarcinoma (COAD), esophageal carcinoma (ESCA), glioblastoma multiforme (GMB), head and neck squamous cell carcinoma (HNSC), kidney chromophobe (KICH), kidney renal clear cell carcinoma (KIRC), kidney renal papillary cell carcinoma (KIRP), liver hepatocellular carcinoma (LIHC), lung squamous cell carcinoma (LUSC), rectum adenocarcinoma Esophageal carcinoma (READ), stomach adenocarcinoma (STAD), thyroid carcinoma (THCA) and uterine corpus endometrial carcinoma (UCEC). Paired *t* test showed STC2 is much higher in tumor samples of bladder urothelial carcinoma (BLCA), COAD, and other types of cancer (Figure 1B). We further performed Kaplan-Meier survival of DSS, DFI and PFI in pan-cancer. It is found that high expression of STC2 can predict poor survival of adrenocortical carcinoma (ACC), CESC, ESCA, HNSC, KICH, KIRP, MESO, SARC, THYM and UVM (Figure 1C, Supplementary Figure 1-3).

### Correlation between STC2 expression and genetic alterations status and immune status in pan-cancer

**Figure 2:**
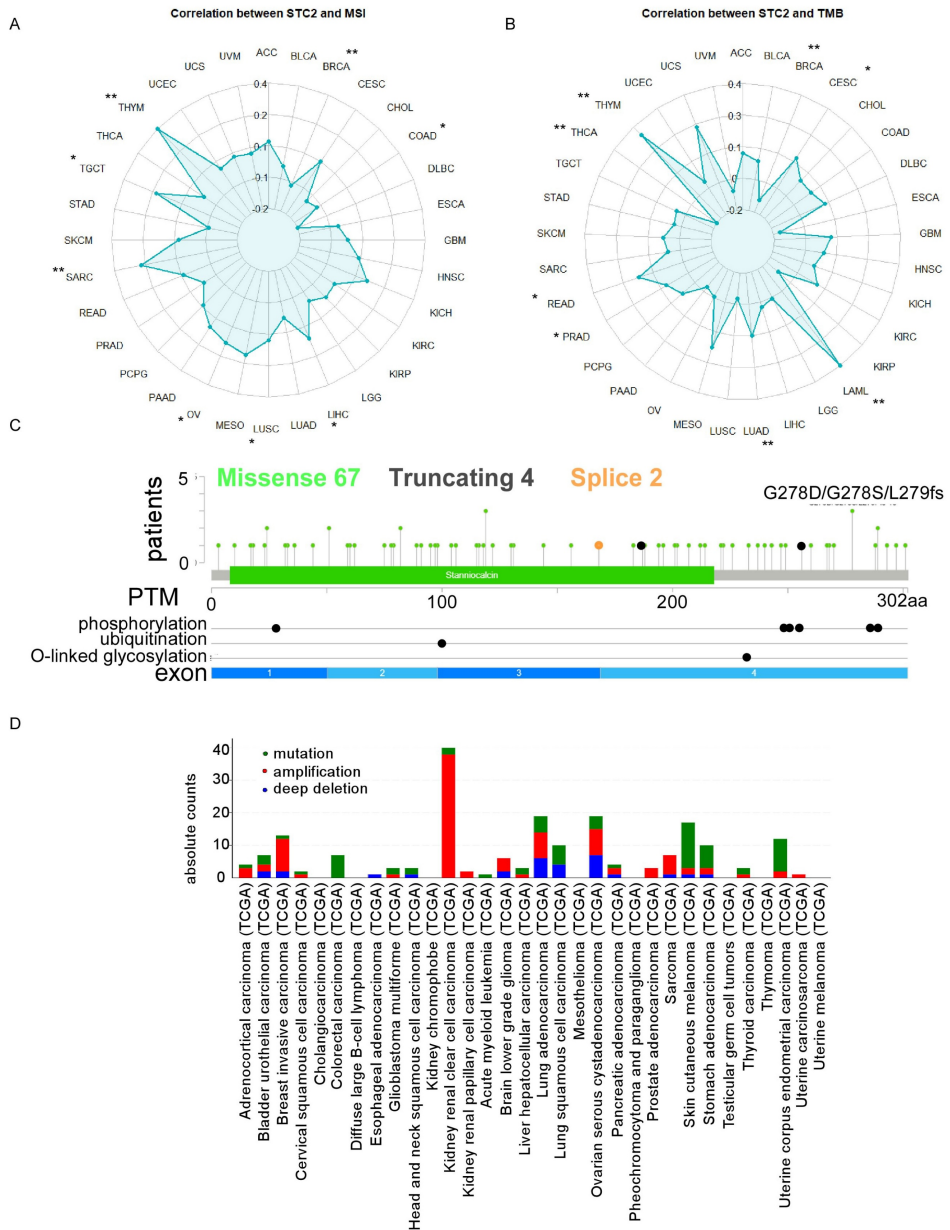
The impacts of STC2 on mutation and immune landscapes across cancer types. A. Radar charts display the correlation of STC2 expression with microsatellite instability (MSI). B. Radar charts display the correlation of STC2 expression with tumor mutational burden (TMB). C. Lollipop plot detailing the mutation spectrum of the STC2 gene across a cohort of patients. The plot shows the positions and frequency of missense, truncating, and splice site mutations along the STC2 protein, with post-translational modifications and glycosylation sites annotated. D. Bar chart showing the frequency of different types of genomic alterations (mutations, amplifications, and deep deletions) in STC2 across a range of cancer tissues. ****, *P* < 0.0001; ***, *P* < 0.001; **, *P* < 0.01; *, *P* < 0.05; ns: not significant.

The STC2 is positively correlated with MSI including LIHC, LUSC, ovarian serous cystadenocarcinoma (OV), SARC, TGCT, THYM and negatively correlated with breast invasive carcinoma (BRCA), and COAD (Figure 2A). The STC2 is positively correlated with TMB including CESC, acute myeloid leukemia (LAML), LUAD, prostate adenocarcinoma (PRAD), READ, THYM and negatively correlated with BRCA, and THCA (Figure 2B). In addition, 67 cases missense mutation, 4 cases truncating mutation, and 2 cases splice mutation was identified in 10976 samples of TCGA pan-cancer analysis and post-transcription modifications of STC2 including phosphorylation, ubiquitination, O-linked glycosylation was characterized through Cbioportal database (Figure 2C). KIRP, OV, BRCA, LUAD and SARC have the highest frequency of amplification; SKCM, STAD, LUSC, COAD and LUAD have the highest frequency of mutation; and LUAD, OV, low grade glioma (LGG), BRCA, and BLCA have the highest frequency of deep deletion (Figure 2D). To further analyze the role of STC2 in cancer immune processes, we further performed a comprehensive analysis between STC2 expression and immune status. STC2 positively correlates with immune scores in BLCA, KICH, KIRC, MESO, pheochromocytoma and paraganglioma (PCPG), PRAD and UVM; but negatively correlates with immune scores in BRCA, CESC, LGG, LUAD, LUSC, TGCT, THCA and UCEC (Supplementary Figure 4A). STC2 is associated with eosinophils, macrophages M0/M1/M2, mast cells activated and NK cells resting in most types of cancer; but negatively associates with B cells memory, CD8 T cells, T cells follicular helper and T cells regulatory across several tumors (Supplementary Figure 4B). STC2 is significantly correlates with immune stimulator genes in most cancers, except BRCA, CESC, LUAD, LUSC, TCGT and THCA; STC2 is significantly correlates with immune inhibitory genes in most cancers, except BRCA, LUSC, TGCT and THCA (Supplementary Figure 5A-5B).

### Data mining of gene expression profiles reveals STC2 was significantly upregulated in CRC across different studies

Since STC2 was significantly upregulated in COAD and associated with poor prognosis in TCGA, we tend to confirm the functional roles of STC2 in other datasets. We mined gene microarray and RNA sequencing data from the Gene Expression Omnibus (GEO), the European Bioinformatics Institute (EMBL-EBI), and The Cancer Genome Atlas (TCGA).

**Figure 3:**
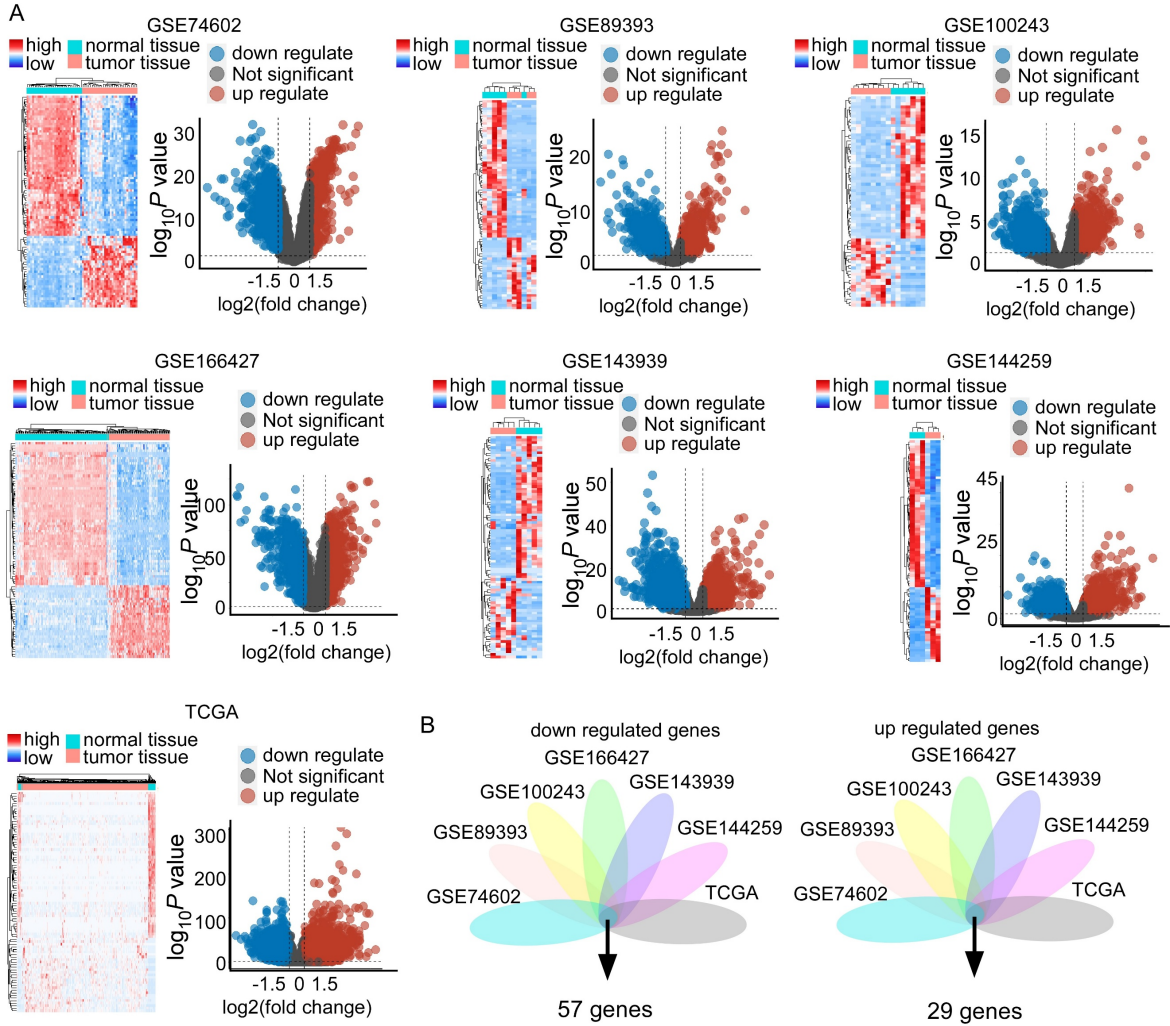
Comparative data mining of differentially expressed gene in colorectal cancer versus non-tumorigenesis tissues indicates STC2 is significantly upregulated across several studies. A. Heatmaps and volcano plots display the expression levels and statistical significance of genes across seven independent datasets (GSE74602, GSE89393, GSE100243, GSE166427, GSE143939, GSE144259, and The Cancer Genome Atlas database). Volcano plots show log2 fold change versus -log10 *P* value for each gene. B. Venn diagrams illustrate the overlap of downregulated and upregulated genes in tumor tissue compared to normal tissue across the datasets. The central intersection identifies 57 commonly downregulated and 29 commonly upregulated genes.

Our analysis included the following datasets: GSE474602, GSE89393, GSE100243, GSE166427, GSE143939, GSE144259, and TCGA datasets. This revealed substantial transcriptional differences between CRC and normal tissues (Figure 3A). Specifically, we identified significant differential expression patterns: 233 upregulated and 312 downregulated genes in GSE474602; 952 upregulated and 1096 downregulated genes in GSE89393; 342 upregulated and 1915 downregulated genes in GSE100243; 425 upregulated and 342 downregulated genes in GSE166427; 1233 upregulated and 2265 downregulated genes in GSE143939; 991 upregulated and 1159 downregulated genes in GSE144259; and 7976 upregulated and 2598 downregulated genes in TCGA (Figure 3A and Supplementary Table 1). Visualization of these changes through heatmaps and volcano plots highlighted distinct expression patterns, with significant upregulation and downregulation of genes in tumor samples compared to normal tissue (Figure 3A). Integrating these datasets revealed a critical overlap of 57 downregulated and 29 upregulated genes across these studies (Figure 3B). STC2 is a top rank gene among the overlap and these solid results indicates the crucial role of STC2 in CRC tumorigenesis (Supplementary Table 1). Besides, we performed GSEA Kyoto Encyclopedia of Genes and Genomes (KEGG) analysis to further elucidate the roles of signaling pathways in colorectal cancer tumorigenesis. The results highlighted several critical pathways significantly implicated in CRC, including DNA replication, homologous recombination, mismatch repair, cell cycle, WNT signaling, aminoacyl-tRNA biosynthesis, and extracellular matrix (ECM) receptor interactions. The top five enriched pathways exhibited normalized enrichment scores (NES) greater than 1.0 and *P* values less than 0.05 (Supplementary Figure 6 and Supplementary Figure 7).

### High expression of STC2 associates with poor prognosis of CRC and the protein level of STC2 is significantly upregulated in CRC

**Figure 4:**
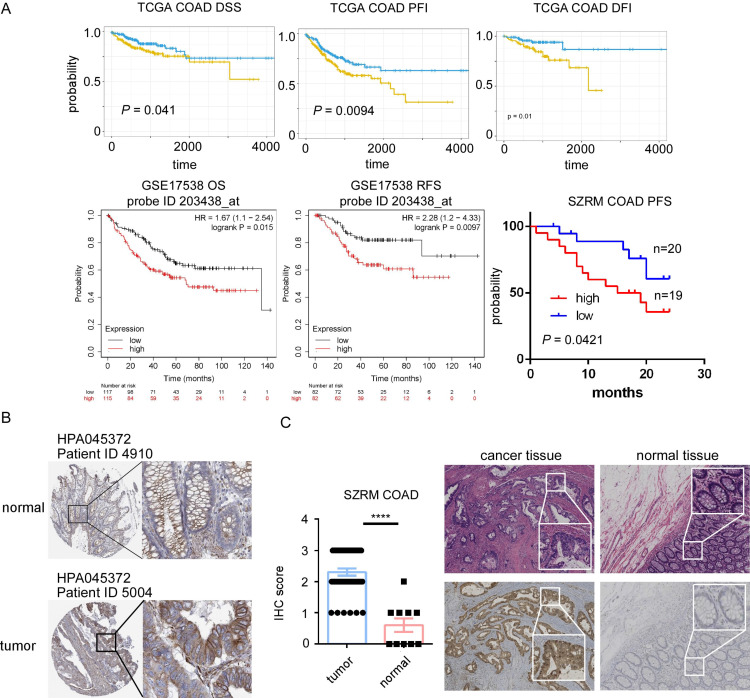
STC2 involved in tumorigenesis of colorectal cancer (CRC). A. Kaplan-Meier survival curves from the TCGA COAD dataset show high expression of STC2 will predict poor DSS, PFI, and DFI. Survival analysis for overall survival (OS) and relapse-free survival (RFS) in GSE17538, highlighting the relationship between high expression of STC2 (probe ID 203438_at) and poor prognosis. High expression of STC2 associated with poor progression free survival in SZRM cohort (n=39). B. Immunohistochemical of STC2 expression in colorectal cancer tissue and normal colon tissue of HPA database (ID 4910 and ID 5004). C.Represent images of immunohistochemical of STC2 expression in colorectal cancer tissue and normal colon tissue in SZRM institution. The IHC score of STC2 was compared between cancer tissues and adjacent non-tumorigenesis colon tissues. ** *P* < 0.01, **** *P* < 0.0001, n.s. not significant).

**Figure 5:**
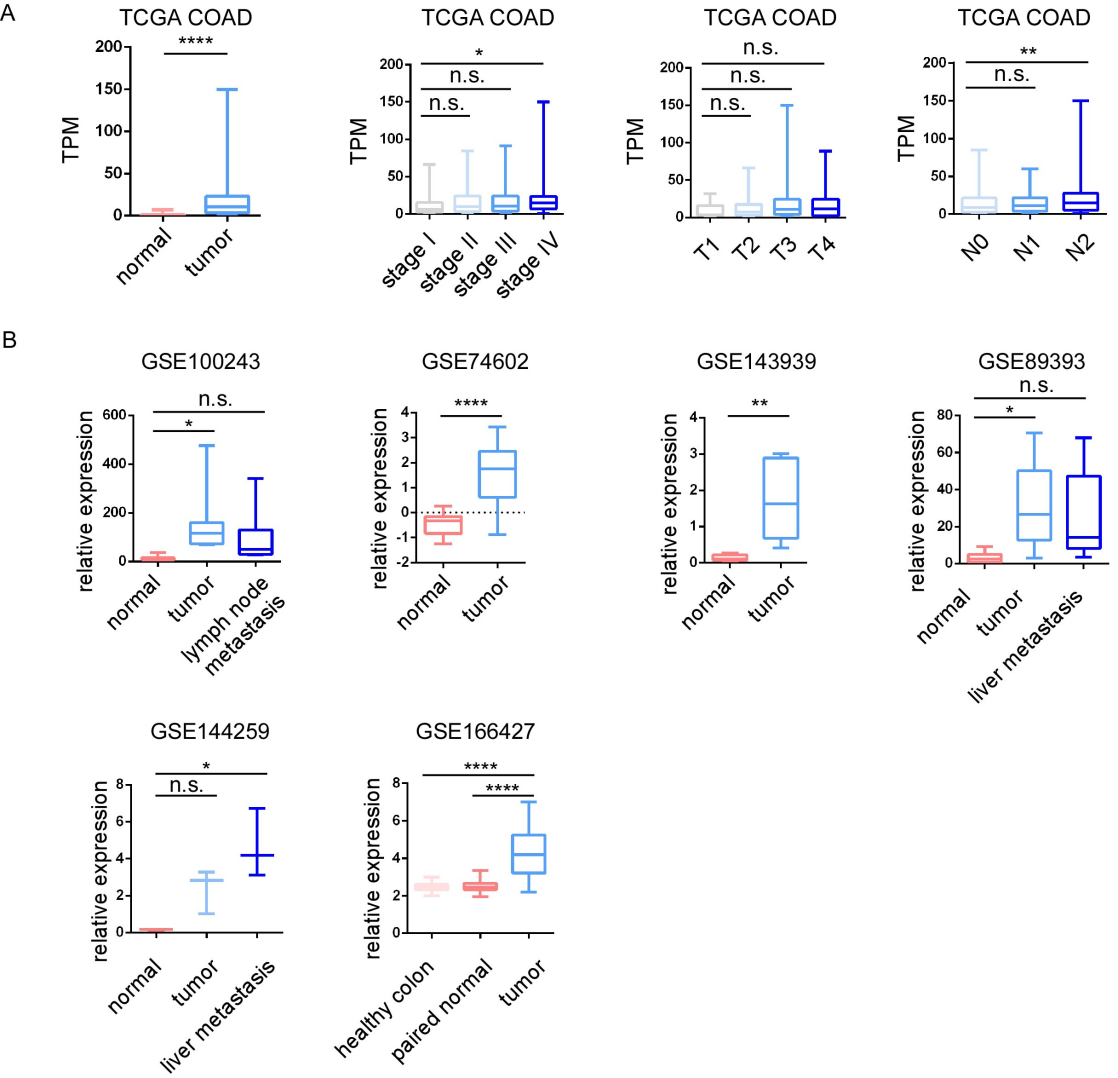
The relative expression of STC2 in colorectal cancer (CRC). A.The relative expression of STC2 was compared between different tumor stages, T stage, N stage and M stage in TCGA COAD. B. The relative expression of STC2 was compared between different tumor stages, T stage, N stage and M stage in GSE100243, GSE74602, GSE143939, GSE89393, GSE144259 and GSE166427. The relative expression of STC2 was compared between cancer tissues and adjacent non-tumorigenesis colon tissues. ** *P* < 0.01, **** *P* < 0.0001, n.s. not significant).

Among 29 upregulated genes, high expression of CBX2, GNG4, PHLDA1, RNF43 and STC2 associated with poor DFI of cancer patients; high expression of CBX2, SULT2B1 and STC2 associated with poor PFI; high expression of STC2 associated with poor DSS; while high expression of MMP1 associated with good DFI; high expression of CDK1 and TMEM97 associated with good PFI; high expression of NFE2L3 associated with good DSS. Among 56 downregulated genes, high expression of AQP8, MT1E and TNFRSF17 associated with good DFI, high expression of AQP8, EDN3, HHLA2, KLF4, MT1G, SLC16A9 and TNFRSF17 associated good PFI; high expression of EDN3 associated with good DSS; while high expression of SLC17A4 associated with poor DFI; high expression of STMN2 associated with poor PFI; and high expression of TRPM6 associated with poor DFI. STC2 was identified as a key prognostic gene, since STC2 is significantly upregulated in CRC samples and significantly predicts poor prognosis of PFI DFI and DSS in TCGA cohort (Figure 4A). In addition, high expression of STC2 was found to associate with poor overall survival and short recurrence free survival in GSE17538, and associate with short progression free survival in our center (Figure 4A). Through HPA and our cohort, IHC staining of the STC2 protein levels of CRC samples showed that STC2 is much higher in colon tissue samples than that in normal colon tissue (Figure 4B-4C). In addition, through TCGA, GSE100243, GSE74602, GSE143939, GSE89393, GSE144259 and GSE166427, it is found that STC2 is significantly expressed by tumor samples and the relative mRNA level of STC2 is significantly higher in stage IV, lymph node metastasis and organ metastasis samples (Figure 5A-5B).

### Knockdown of STC2 leads to impaired cell migration, invasion and proliferation in CRC cells

**Figure 6:**
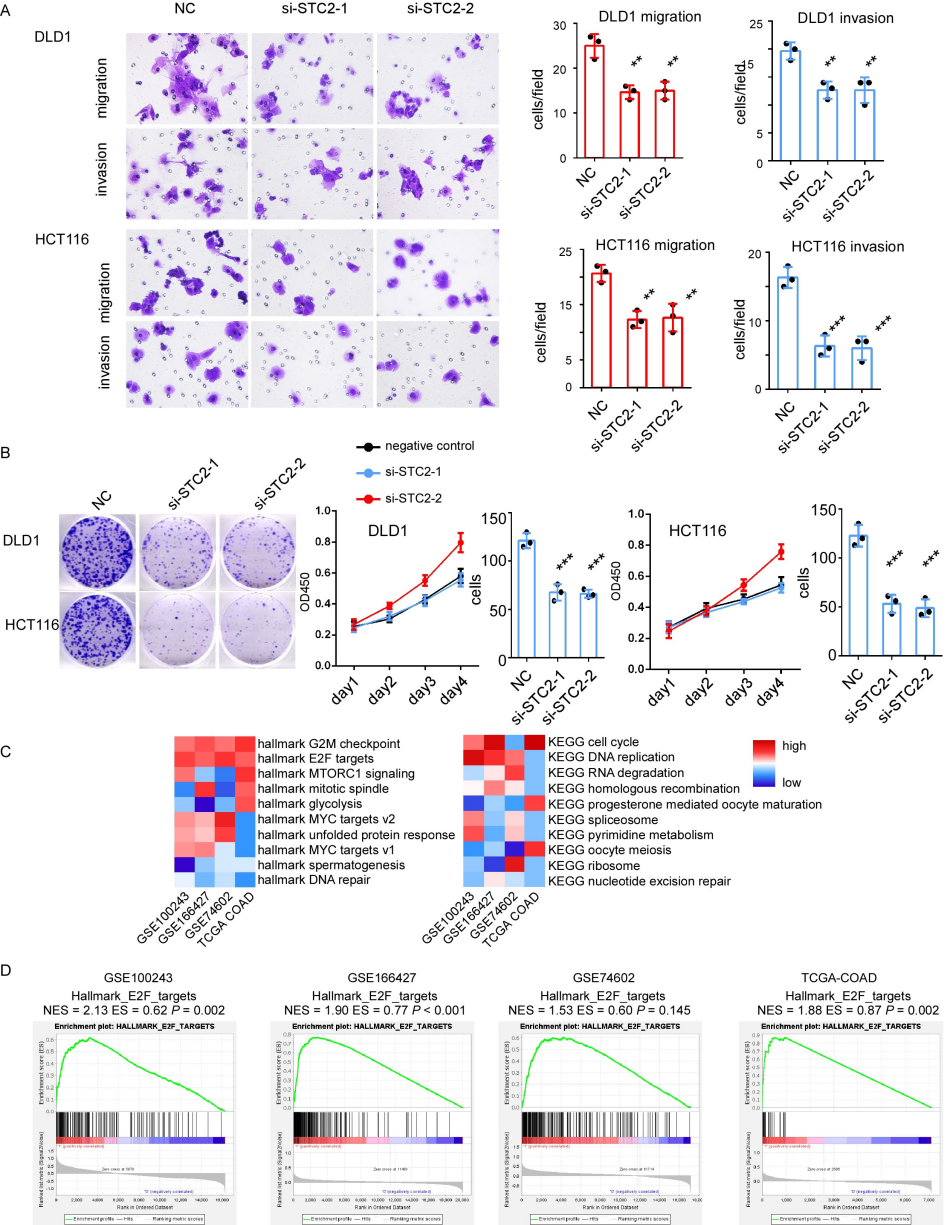
Knockdown of STC2 impacts on colorectal cancer (CRC) cell proliferation, migration and invasion. A. Represent images of migration ability and invasion ability of CRC cell lines DLD1 and HCT116 after STC2 knockdown. Histogram of three independent experiments of migration and invasion assays after STC2 knockdown. B. The proliferation ability of DLD1 and HCT116 determined by the CCK8 assay and colony formation after STC2 knockdown. C. GSEA hallmark and GSEA KEGG pathway enrichment of high STC2 samples in different cohorts. D GSEA hallmark E2F targets of high STC2 samples in different cohorts. n.s., not significant, *P* > 0.05; * *P* < 0.05; ** *P* < 0.01; **** *P* < 0.0001.

The role of STC2 in CRC progression was further determined. Firstly, we employ Cancer Cell Line Encyclopedia datasets to study the relative expression of STC2 in different types of cancer cell lines (Supplementary Figure 8A). STC2 is highly expressed in most CRC cancer cell lines. Two siRNAs targeting STC2 were used to create STC2-silencing cell models. Transfection of STC2 siRNAs significantly leads the reduction of cancer cell function. Knockdown of STC2 will significantly decrease migration ability and invasion capacity of DLD1 and HCT116 in transwell assay (Figure 6A). The CCK8 assay indicated that the silencing of STC2 significantly inhibited DLD1 and HCT116 proliferation (Figure 6B). The colony formation ability of DLD1 and HCT116 was decreased after STC2 siRNAs transfection (Figure 6B). This indicates that STC2 is essential for CRC cell proliferation. However, silence of STC2 in normal colorectal epithelial cells slightly inhibits cell colony formation ability and transwell migration ability with *P* value not significant (Supplementary Figure 8B-8C). These results indicate STC2 is a potential oncogene as experiments *in vitro* suggest that STC2 promotes CRC cell proliferation, maintains CRC cell viability, associates with CRC cell migration and invasion. To further explore the downstream signaling pathways of STC2, we performed GSEA analysis in cohorts with large sample sizes. Through GSEA, we found that GSEA hallmark such as G2M checkpoints, E2F targets, MYC targets, MTORC1 signaling were enriched and GSEA KEGG such as cell cycle, DNA replication, RNA degradation, homologous recombination were enriched (Figure 6C-6D). Furthermore, minichromosome maintenance 10 replication initiation factor (MCM10), an E2F target^18^, was significantly associated with the expression of STC2 (data not show). This suggested E2F signaling pathway is potential downstream of STC2.

## Discussion

Our comprehensive data mining and analysis of gene expression profiles across multiple datasets have provided significant insights into the molecular underpinnings of colorectal cancer (CRC) progression. The identified patterns of gene expression alterations highlight the complexity of CRC pathogenesis and suggest several potential biomarkers and therapeutic targets, with particular emphasis on the role of STC2. STC2, a secreted glycoprotein implicated in various physiological processes, including calcium and phosphate homeostasis, has emerged as a pivotal player in physiology and pathology^5^, ^6^. Some studies had revealed the important roles of STC2 in cancer progression and oncogenesis in breast cancer and hepatocellular carcinoma^11^, ^19^. Few studies have covered the roles of STC2 in colorectal cancer.

Our data mining across multiple datasets, including GEO, EMBL-EBI, and TCGA, has revealed significant transcriptional differences between CRC and normal tissues, with a critical overlap of differentially expressed genes. The substantial transcriptional differences between CRC and normal tissues that we observed reinforce the notion that CRC development is driven by the dysregulation of specific gene sets involved in critical biological processes. Notably, STC2 was consistently upregulated across these datasets, suggesting its integral role in CRC pathophysiology. The consistent identification of significant upregulated STC2 across different datasets, including GSE474602, GSE89393, and others, underlines the robustness of our analysis methods and the potential reliability of these findings. Besides, the enrichment of cancer-associated KEGG signaling pathways in CRC further underscores the complexity of CRC tumorigenesis^20^, ^21^. Consistently, pathways such as DNA replication^22^, ^23^, cell cycle^24^, ^25^, and the extracellular matrix (ECM) receptor interactions^26^, ^27^, which are crucial for tumor growth and metastasis, were significantly implicated.

Moreover, our analysis highlighted STC2's prognostic significance, with its overexpression correlating with poor disease-free interval (DFI), progression-free interval (PFI), and disease-specific survival (DSS) in CRC patients. This prognostic value extends to pan-cancer analysis, where STC2 expression levels were significantly elevated in various cancer types, particularly in advanced stages and lymph node metastasis samples. This is in line with studies indicating STC2's involvement in CRC progression that STC2 is an independent prognostic marker of overall survival in CRC and knockdown of STC2 can inhibit CRC cell migration^13^, ^28^. Such a broad impact suggests that STC2 may serve as a universal marker for aggressive cancer phenotypes and could be a target for therapeutic intervention.

Interestingly, STC2's expression was also associated with genetic alterations, including microsatellite instability (MSI) and tumor mutational burden (TMB), highlighting its potential involvement in genomic instability, a hallmark of cancer. The association with high MSI and TMB in certain cancers suggests that STC2 could be involved in the response to genomic stress^23^, ^29^. Moreover, the relationship between STC2 expression and immune infiltration underscores its possible role in modulating the tumor immune microenvironment, which is a crucial aspect of tumor progression and response to therapy^30^. These results across different cancer types further emphasize the gene's role in the mutagenic processes that drive tumorigenesis. The correlation between STC2 expression and patient survival events in pan-cancer analysis is particularly interesting. High STC2 expression predicted poor survival outcomes in several cancer types, reinforcing its prognostic relevance. This oncogenic role is further supported by the gene's positive correlation with immune processes, such as immune infiltration scores and abundance scores of various immune cells. STC2's association with immune stimulatory and inhibitory genes suggests it may influence the tumor microenvironment, potentially affecting immune surveillance and response to therapy.

Given the significant roles of STC2 in CRC and potentially other cancers, targeting STC2-related pathways might offer new therapeutic opportunities. The association of STC2 with poor prognosis and aggressive cancer features suggests that STC2 inhibitors could potentially improve patient outcomes. Additionally, the involvement of STC2 in key regulatory pathways and its impact on the immune environment of tumors presents a compelling case for the development of combination therapies that target both STC2 and immune checkpoints.

In conclusion, our study, along with existing research, positions STC2 as a gene of considerable interest in cancer biology. Its consistent upregulation in CRC and other cancers, coupled with its prognostic significance and association with key genetic and immune processes, underscores its potential as a biomarker and therapeutic target. Future research should focus on elucidating the precise mechanisms by which STC2 contributes to cancer progression and exploring its utility in clinical applications.

## Conclusion

In conclusion, our findings highlight the importance of STC2 as a biomarker and potential therapeutic target in CRC. The insights gained from this study not only enhance our understanding of CRC pathogenesis but also suggest broader implications for the treatment of other cancers. Future studies should focus on validating these potential targets and exploring the mechanistic roles of STC2 in cancer biology to fully exploit its therapeutic potential. The integration of genomic, transcriptomic, and clinical data will be pivotal in advancing our understanding and treatment of CRC and potentially other malignancies where STC2 plays a critical role.

## Supplementary Material

Supplementary figures and table.
