# Supplementary material for: Stanniocalcin-2 significantly promotes colorectal cancer progression by regulating cancer cell proliferation and invasion
Source: J Cancer. 2025 Jun 12;16(8):2737–49. doi: 10.7150/jca.101892 (PMC12170999; doi:10.7150/jca.101892)
Supplement: Supplementary file 1 — Supplementary figures and table. [file jcav16p2737s1.pdf]

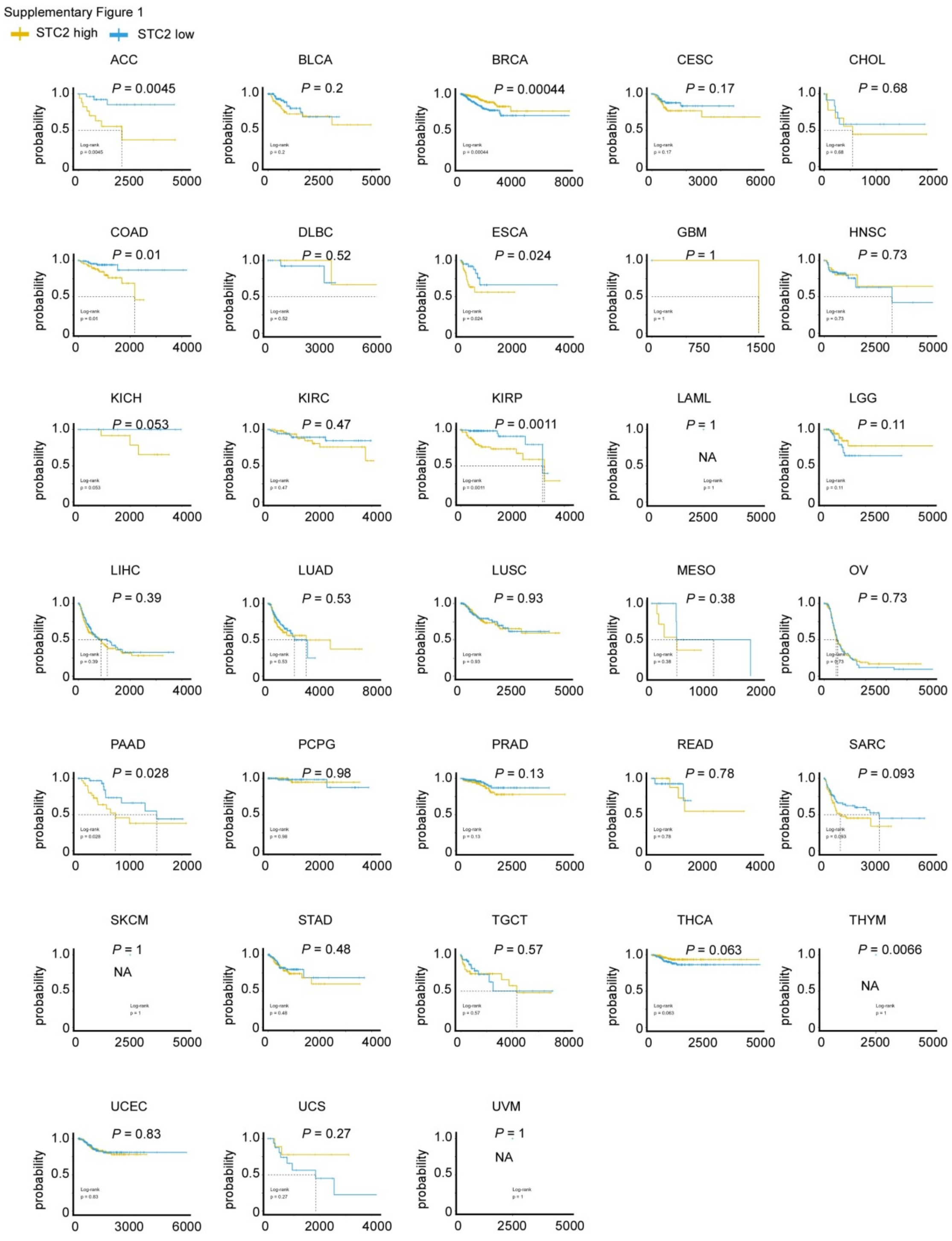

Supplementary Figure 1. Kaplan-Meier survival curves of DFI from the TCGA pan-cancer analysis.

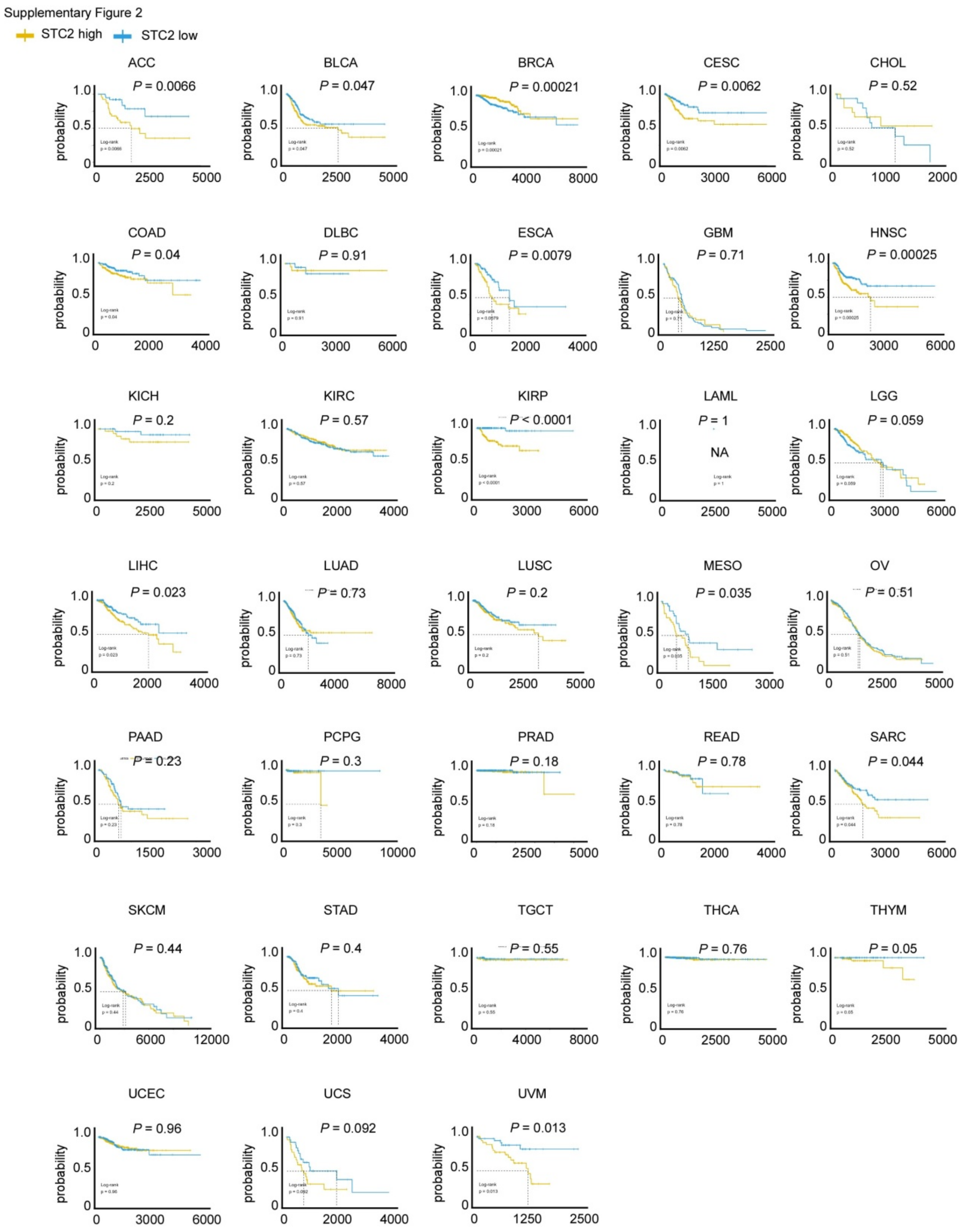

Supplementary Figure 2. Kaplan-Meier survival curves of DSS from the TCGA pan-cancer analysis.

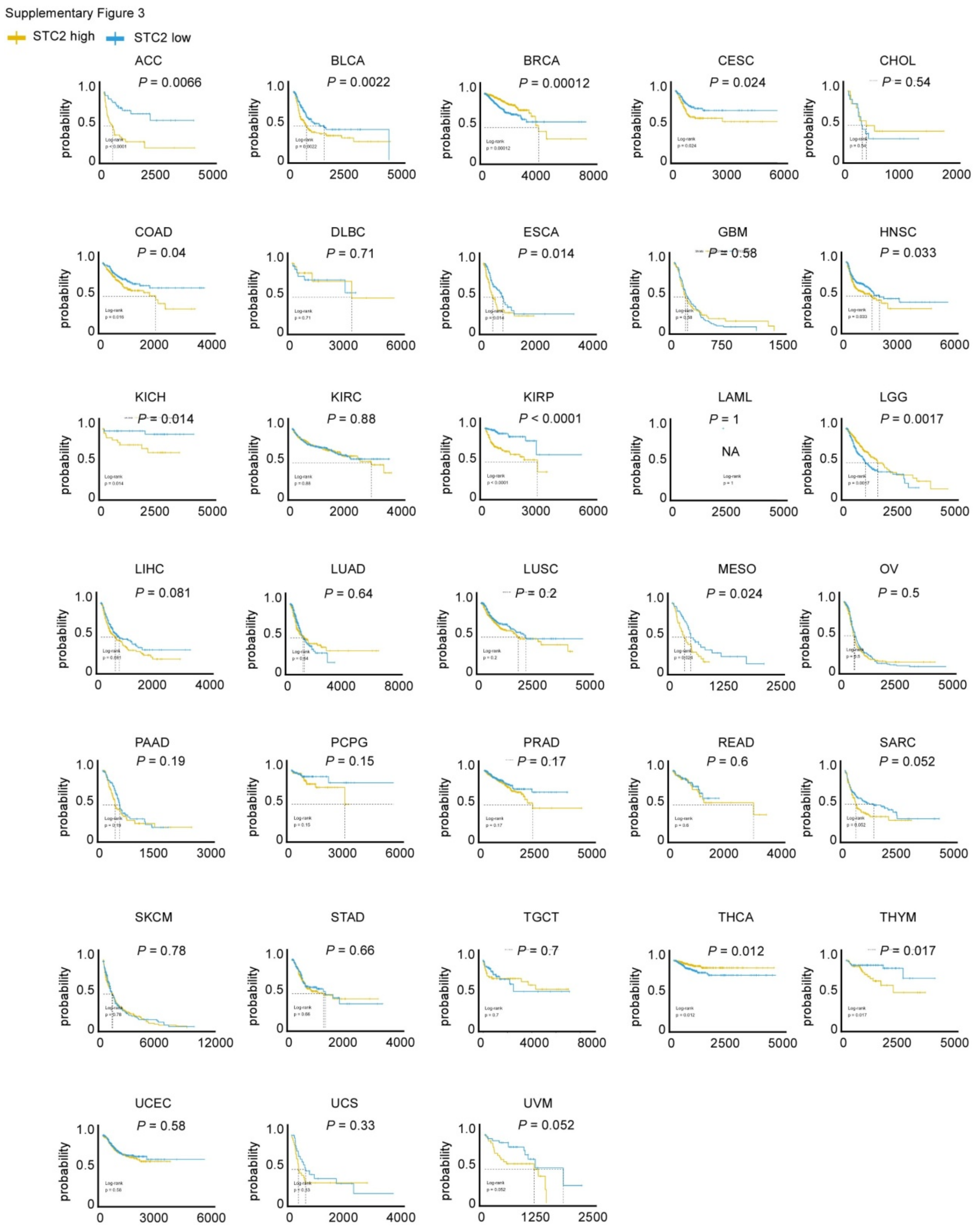

Supplementary Figure 3. Kaplan-Meier survival curves of PFI from the TCGA pan-cancer analysis.

Supplementary Figure 4

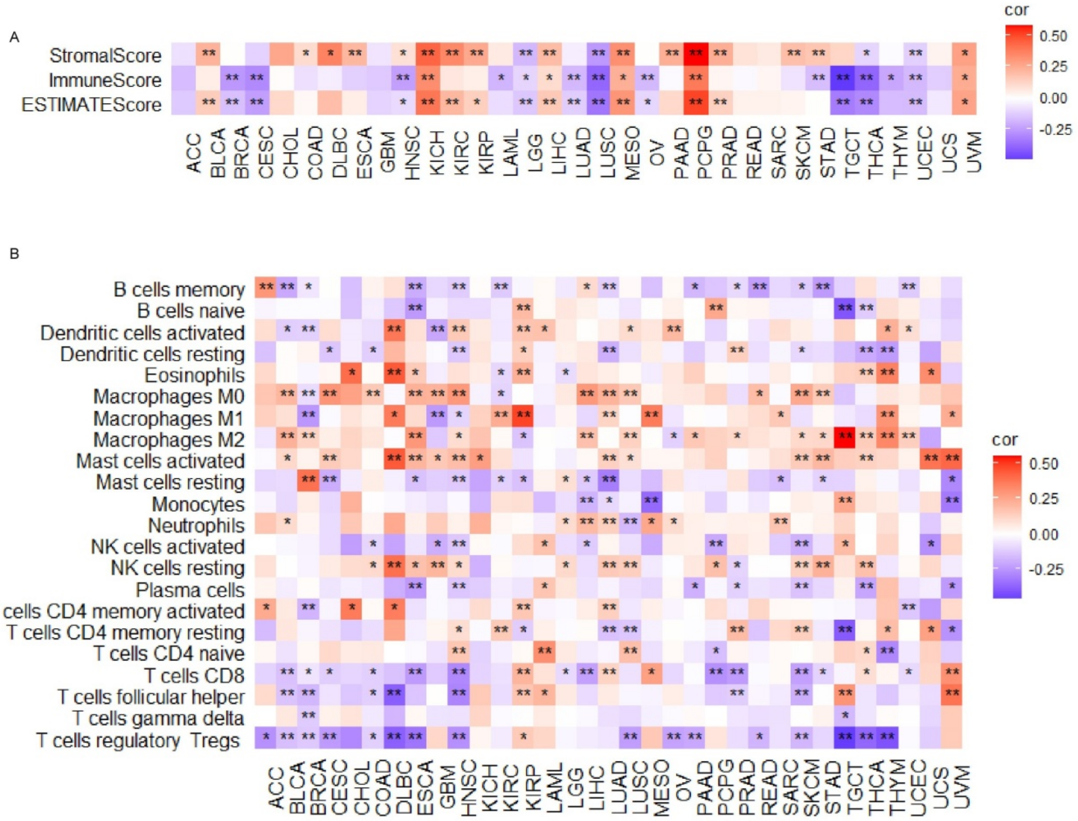

Supplementary Figure 4. A. Heatmap depicting the correlation coefficients between stromal, immune, and ESTIMATE scores and various cancer types. B. Heatmap illustrates the correlation of abundance of different immune cell types with the listed cancer types. . ****, $P < 0.0001$; ***, $P < 0.001$; **, $P < 0.01$; *, $P < 0.05$; n.s., not significant.

Supplementary Figure 5

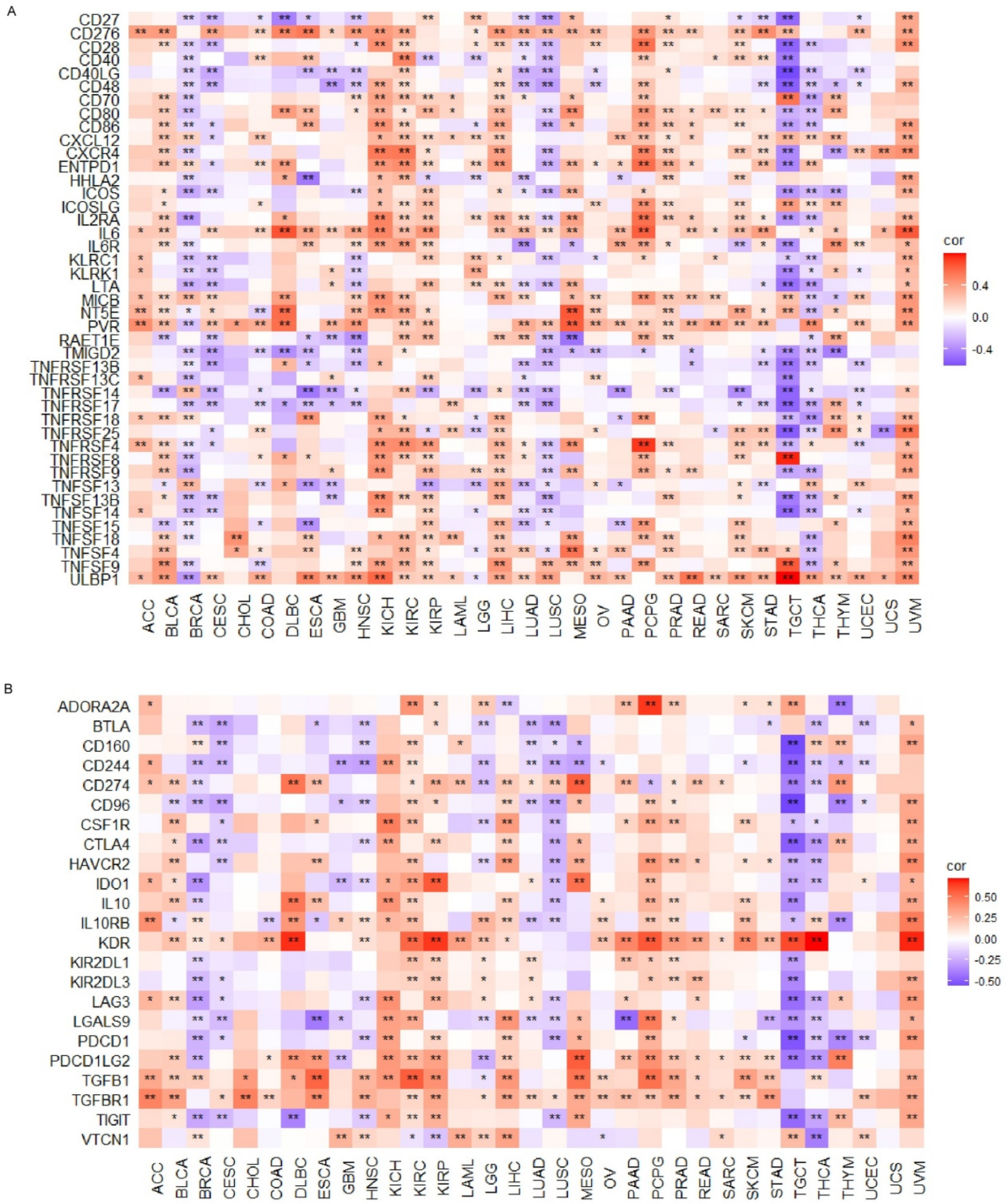

Supplementary Figure 5. A. Heatmap shows the correlation between the expression of STC2 and immune stimulator genes in different cancer types. B. Heatmap shows the correlation between the expression of STC2 and immune inhibitory genes in different cancer types.

Supplementary Figure 6

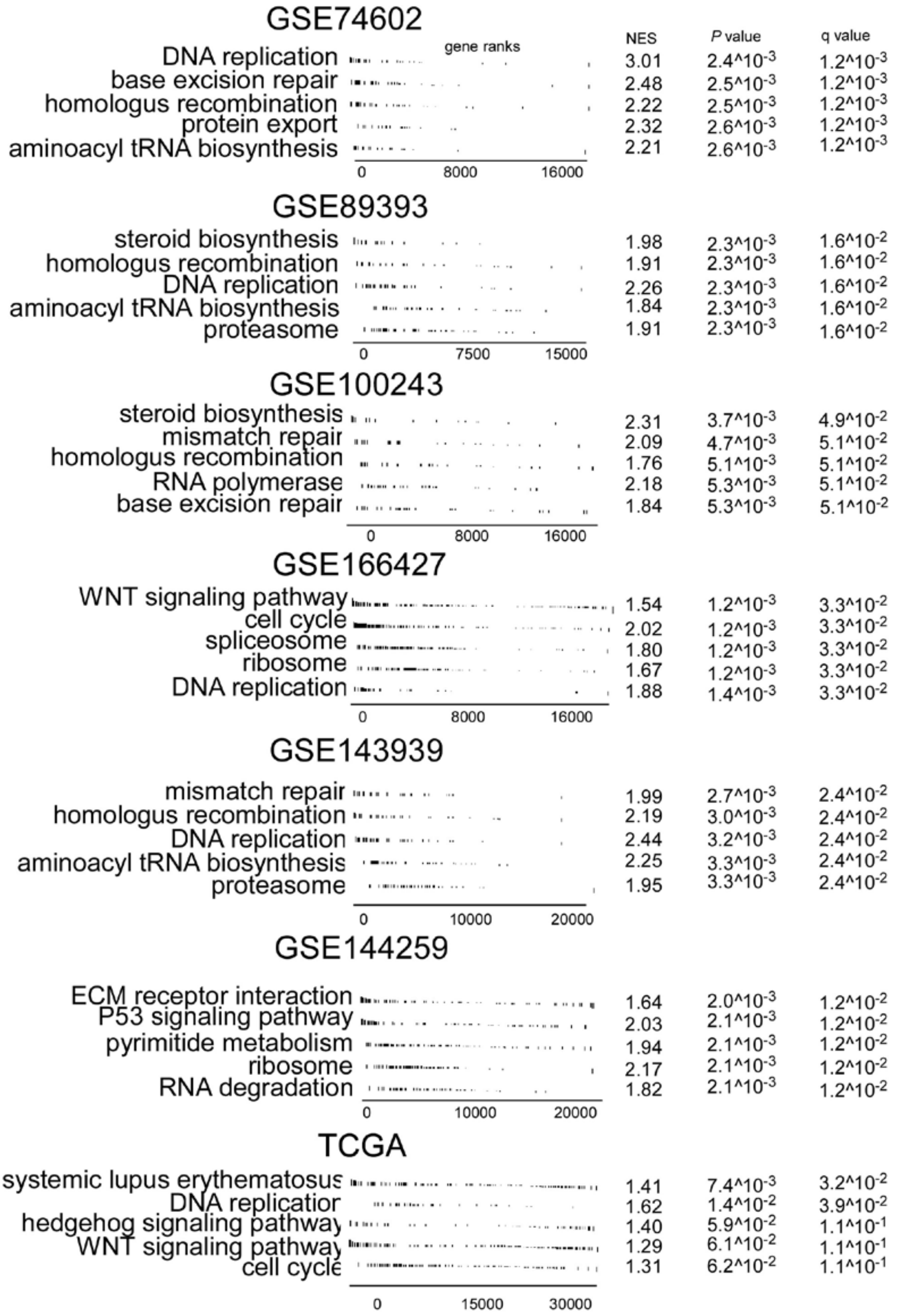

Supplementary Figure 6. Positive enrichment sets of Kyoto Encyclopedia of Genes and Genomes (KEGG) enrichment analysis of gene expression in colorectal cancer versus normal tissues across multiple datasets. Gene sets from GSE74602, GSE89393, GSE100243, GSE166427, GSE143939, GSE144259, and the TCGA are analyzed for pathway enrichment. The pathways are ranked based

on Normalized Enrichment Score (NES), *P* value and false discovery rate (FDR) q-values. Dotted lines across the enrichment plots indicate the threshold for statistical significance. Pathways with a *P* value less than 0.05 are considered significantly enriched and are indicated with asterisks.

Supplementary Figure 7

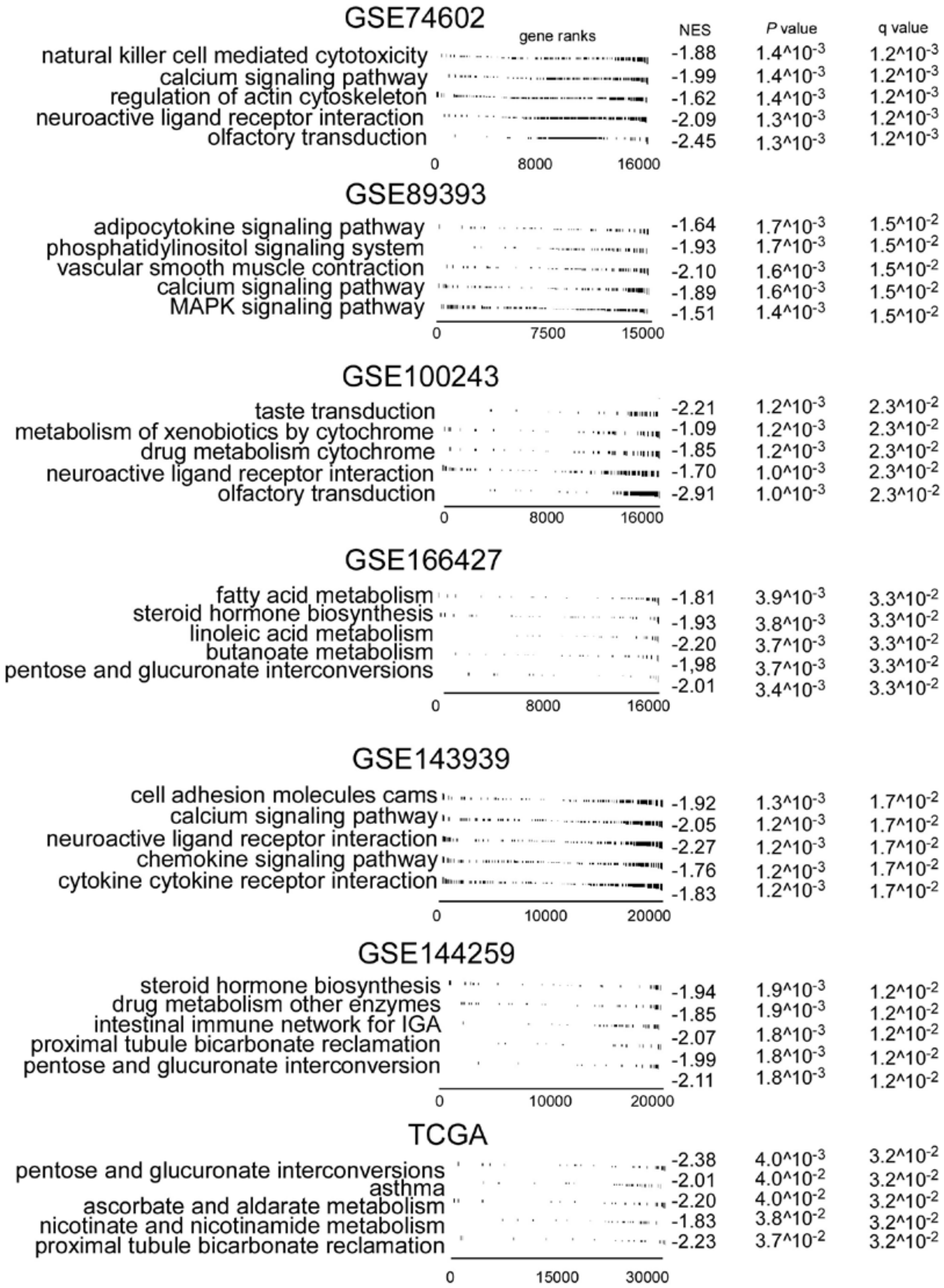

Supplementary Figure 7. Negative enrichment sets of Kyoto Encyclopedia of Genes and Genomes (KEGG) enrichment analysis of gene expression in colorectal cancer versus normal tissues across multiple datasets. Gene sets from GSE74602, GSE89393, GSE100243, GSE166427, GSE143939, GSE144259, and the TCGA are analyzed for pathway enrichment. The pathways are ranked based on Normalized Enrichment Score (NES), *P* value and false discovery rate (FDR) q-values. Dotted lines across the enrichment plots indicate the threshold for statistical significance. Pathways with a

P value less than 0.05 are considered significantly enriched and are indicated with asterisks.

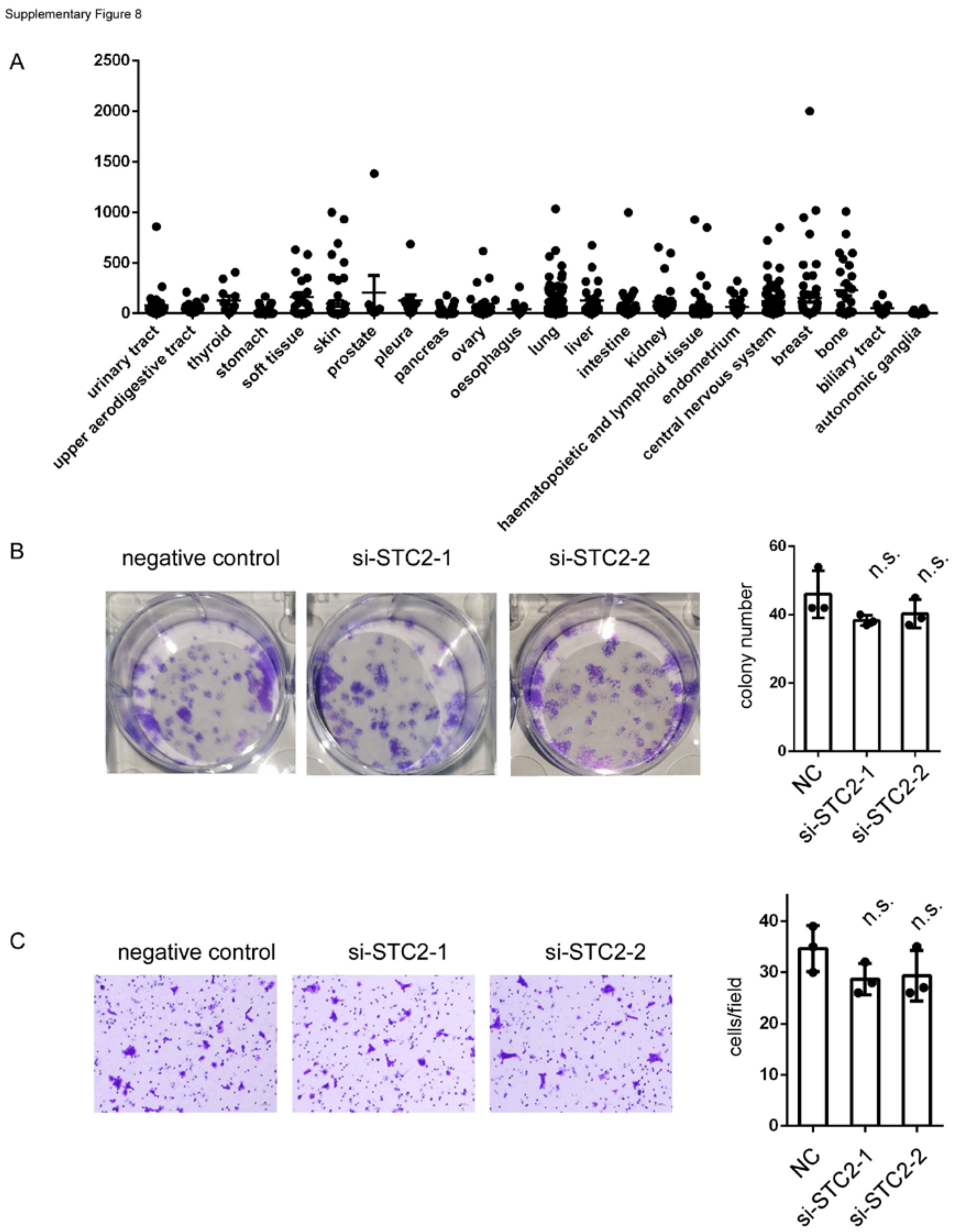

Supplementary Figure 8. The roles of STC2 in HIEC6 normal colorectal cells. A. The relative expression of STC2 in CCLE database. B. Colony formation ability of HIEC6 after STC2 siRNA transfection. C. Transwell migration ability of HIEC6 after STC2 siRNA transfection. n.s., not significant.

Supplementary Table 1. Differentially expressed genes of 7 independent datasets (GSE74602, GSE89393, GSE100243, GSE166427, GSE143939, GSE144259, and TCGA-COAD).

GEO number	Gene ID	Gene name	Adjust P value	*P* value	Log2FC
GSE74602	ILMN_1691884	STC2	2.08E-11	1.22E-12	2.0051247
GSE89393	8614	STC2	5.97E-04	2.05E-05	3.171764
GSE100243	8614	STC2	4.92E-08	2.45E-11	3.342787
GSE166427	11721436_a_at	STC2	3.72E-41	2.35E-42	1.78
GSE143939	8614	STC2	8.77E-15	8.62E-17	3.751376
GSE144259	8614	STC2	8.11E-08	3.78E-10	3.852166
TCGA	ENSG00000113739.10	STC2	1.19E-54	1.46E-52	3.594413
